# Increased expression of lncRNA FTH1P3 predicts a poor prognosis and promotes aggressive phenotypes of laryngeal squamous cell carcinoma

**DOI:** 10.1042/BSR20181644

**Published:** 2019-06-18

**Authors:** Haozhan Yuan, Hong Jiang, Yanting Wang, Yameng Dong

**Affiliations:** 1 Department of Otolaryngology, The First People’s Hospital of Xianyang, Xianyang 712000, People’s Republic of China; 2 Department of Otolaryngology, The Nuclear Industry 215 Hospital of Shaanxi Province, Xianyang 712000, People’s Republic of China

**Keywords:** biomarker, cell, FTH1P3, LSCC, prognosis

## Abstract

Laryngeal squamous cell cancer (LSCC) is a highly aggressive malignancy in the head and neck region. Recent studies have shown that long noncoding RNAs (lncRNAs) are novel transcripts that play an important role in the progression of LSCC. However, the overall pathophysiological regulation of lncRNAs to LSCC is largely unknown. The present study aimed to determine the clinical significances of lncRNA ferritin heavy chain 1 pseudogene 3 (FTH1P3) and to identify its potential roles in LSCC. Quantitative real-time PCR (qRT-PCR) showed that FTH1P3 expression was significantly up-regulated in LSCC tissues than that in non-neoplastic tissues. High FTH1P3 expression was positively correlated with the poor differentiation, high T classification, positive lymph node metastasis, and advanced clinical stage. Overall survival analysis showed that high levels of FTH1P3 predicted a poor prognosis in LSCC patients. Moreover, elevated expression of FTH1P3 was found to increase LSCC cell proliferation, migration and invasion, and to inhibit cell apoptosis, Conversely, knockdown of FTH1P3 suppressed LSCC cell proliferation, migration and invasion, and induced cell apoptosis. In addition, overexpression of FTH1P3 resulted in an increase in cells in S phase and a decrease in cells in G0/G1 phase, whereas inhibition of FTH1P3 did the opposite effects. Taken together, these results suggested that increased expression of FTH1P3 predicts a poor prognosis and promotes aggressive phenotypes of LSCC by regulating cell proliferation, migration, invasion, apoptosis, and cell cycle, indicating FTH1P3 may serve as a promising therapeutic biomarker for the treatment of LSCC.

## Introduction

Laryngeal cancer is one of the most aggressive cancers of the head and neck, and more than 95% of laryngeal cancers are laryngeal squamous cell carcinoma (LSCC) [1]. Despite recent considerable advances in surgical and oncological treatments, the prognosis of LSCC patients remains unfavorable [2,3]. Patients with metastasis and invasion of LSCC have much worse prognosis, with a 5-year survival rate of approximately 60% [4]. Frequent recurrence and metastasis are the main factors resulting in treatment failure. Therefore, it is urgent to determinate the underlying mechanisms of LSCC and to identify novel effective therapeutic strategies for the treatment of LSCC.

Long noncoding RNAs (lncRNAs) are defined as RNA transcripts larger than 200 nucleotides (nt) and cannot be translated into proteins [5]. Accumulating evidences have demonstrated that lncRNAs play important regulatory roles in a variety of biological processes, including cell proliferation, differentiation, migration, cell cycle, and apoptosis [6,7]. Notably, a variety of lncRNAs have been found to be dysregulated and functioned as oncogenes or tumor suppressor genes in many human cancers, such as renal cancer [8], lung cancer [9], hepatocellular carcinoma [10], and osteosarcoma [11]. For example, overexpression of lncRNA MALAT1 (metastasis-associated lung adenocarcinoma transcript 1) promotes aggressive phenotypes of renal cell carcinoma by regulating cell proliferation and invasion [12]. LncRNA OIP5-AS1 (OIP5 antisense RNA 1) overexpression leads to poor prognosis and acts as a growth-promoting lncRNA in lung cancer [13]. Up-regulation of lncRNA PTTG3P (pituitary tumor-transforming 3, pseudogene) promotes tumor cell growth and metastasis in hepatocellular carcinoma [14]. Down-regulation of lncRNA GAS5 (growth arrest specific 5) represses osteosarcoma cells growth and metastasis in osteosarcoma [15]. Although large numbers of lncRNAs have been annotated, the potential functions of lncRNAs in LSCC progression still require further investigation.

Ferritin heavy chain 1 pseudogene 3 (FTH1P3, NR_002201) is a kind of lncRNA, and locates in 2p23.3 with 954 nucleotides in length. Previous literatures have reported that FTH1P3 is up-regulated in oral squamous cell carcinoma (OSCC) and uveal melanoma [16,17]. Further studies have found that FTH1P3 facilitates cell growth and invasion through regulating microRNA-224-5p in OSCC and uveal melanoma [16,17]. After that, lncRNA microarray analysis has shown that FTH1P3 expression is up-regulated in LSCC [18]. However, the physiological roles of FTH1P3 in LSCC are yet poorly understood. The present study was undertaken to verify the expression of FTH1P3 in LSCC tissues, and to assess its clinical significance and roles in the progression of LSCC. The findings suggested that elevated expression of FTH1P3 predicts a poor prognosis and promotes aggressive phenotypes of LSCC by regulating cell proliferation, migration, invasion, apoptosis, and cell cycle. The study shows a significant step forward in understanding the importance of FTH1P3 in LSCC, and provides a new insight concerning the roles of FTH1P3 in the progression of LSCC.

## Materials and methods

### Patient samples

Fresh LSCC tissues and adjacent non-neoplastic tissues (5 cm away from the tumor boundary) were obtained from 40 cases of LSCC patients subjected to surgical resection at the Department of Otolaryngology, The First People’s Hospital of Xianyang (Xianyang, China) between February 2008 and August 2013. The patients had not received radiotherapy and/or chemotherapy before admission. All tissue samples were evaluated by histopathologically examination in our hospital according to the 8th edition of American Joint Committee on Cancer (AJCC) tumor-node-metastasis (TNM) staging system [19]. Samples were quickly frozen in liquid nitrogen and subsequently stored at −80°C for further experiments. The clinicopathological characteristics of the patients were collected and summarized in Table 1. The present study was approved by the Institutional Ethics Committee of The First People’s Hospital of Xianyang, and informed consent forms were obtained from each participant.

**Table 1:** Relationship between FTH1P3 expression and clinicopathologic parameters of LSCC patients

Characteristic	No.	FTH1P3 expression level		*P* value
	(*n* = 40)	High (*n*=18)	Low (*n*=22)	
Gender				0.622
Female	15	6	9	
Male	25	12	13	
Age (years)				0.131
<55	17	10	7	
≥55	23	8	15	
Primary location				0.324
Supraglottic	21	11	10	
Glottic	19	7	12	
Differentiation				0.035*
High	12	2	10	
Moderate + poor	28	16	12	
T classification				0.001*
T1 + T2	14	1	13	
T3 + T4	26	17	9	
Lymph node metastasis				0.046*
No	14	3	11	
Yes	26	15	11	
Clinical stage				0.016*
I + II	13	2	11	
III + IV	27	16	11	

T, tumor. *P<0.05.

### Cell culture and cell transfection

Human LSCC cell lines (Hep-2 and TU212) were purchased from the Cell Institute of Chinese Academy of Science (Shanghai, China). Hep-2 and TU212 cells were cultured in RPMI 1640 medium (Gibco, Carlsbad, CA, U.S.A.) with 10% fetal bovine serum (FBS; Gibco, Carlsbad, CA, U.S.A.) in a humidified incubator at 37°C with 5% CO_2_ atmosphere, and supplemented with 1% penicillin/streptomycin.

The human FTH1P3 shRNA (short hairpin RNA) lentiviral vector (FTH1P3 shRNA) and the control shRNA lentiviral vector (control shRNA), and the FTH1P3 overexpressed lentiviral vector (FTH1P3 overexpressed vector) and the empty overexpressed lentiviral vector (empty overexpressed vector) were designed and constructed by GenePharma Co., Ltd (Suzhou, China). The sequences of FTH1P3 shRNA were as follows: 5′-CACCGCCAGCCCTCCGTCACCTCTTCGAAAAGAGGTGACGGAGGGCTGGC-3′ (top strand), 5′-AAAAGCCAGCCCTCCGTCACCTCTTTTCGAAGAGGTGACGGAGGGCTGGC-3′ (bottom strand).

For cell transfection, Hep-2 and TU212 cells were plated in 12-well plates (5 × 10^4^ cells/well) and cultured overnight at 37°C in a humidified incubator. Lentiviruses were diluted in 0.2 ml RPMI 1640 medium containing polybrene (8 mg/ml), and were added into cells and incubated for 1 h in a humidified incubator at 37°C. Then, cells were incubated with 0.3 ml fresh prepared RPMI 1640 medium containing polybrene for 24 h. Finally, the cells were cultured with fresh RPMI 1640 medium and 10% FBS for 48 h. After that, the cells were collected for the following *in vitro* cell experiments.

### Quantitative real-time PCR analysis

Total RNA from LSCC tissues and cells were isolated by using TRIzol solution (Invitrogen, Carlsbad, CA, U.S.A.). The RNA was reversely transcribed into cDNA (Complementary DNA) by using a PrimeScript RT Reagent Kit (Takara, Dalian, China), according to the manufacturer’s instructions. Quantitative real-time PCR (qRT-PCR) assay was performed to detect FTH1P3 expression by using a SYBR® Premix Ex Taq™ II kit according to the manufacturer’s protocols. The primers sequences were GAPDH (glyceraldehyde-3-phosphate dehydrogenase), sense: 5′-TCAAGAAGGTGGTGAAGCA-3′ and antisense, 5′-AGGTGGAGGAGTGGGTGT-3′; FTH1P3, forward: 5′-CTACGCCTCCTCCATTTA-3′ and reverse: 5′-GCCACCTCGTTGGTTCTA-3′. The conditions of qRT-PCR were as follows: 94°C for 10 min followed by 40 cycles at 94°C for 10 s, 60°C for 30 s, and 72°C for 30 s. The expressions of FTH1P3 was normalized to the expression of GAPDH, and calculated by using 2^−ΔΔ*C*_t_^ method.

### CCK-8 assay

The proliferation ability of Hep-2 cells was determined by using cell count kit-8 (CCK-8) assay. Briefly, the transfected cells were seeded into 96-well plates (5 × 10^3^ cells/well) and cultured for four time points (0, 24, 48, and 72 h). Then, 12 μl CCK-8 solution was added to each well and incubated in a humidified incubator at 37°C for 1 h. The absorbance was detected by using an ELISA microplate reader (Model 680; Bio-Rad, Hercules, CA, U.S.A.) at a wavelength of 450 nm with 630 nm as the reference wavelength.

### Colony formation assay

For colony formation assay, transfected TU212 cells were trypsinized into single cell and seeded in the 6-well plates at a density of 800 cells/well. After 10 days of cell culture, clones that had formed from individual cells were fixed with 4% paraformaldehyde for 20 min and stained with 0.1% crystal violet (Sigma–Aldrich, MO, U.S.A.) for 30 min at 37°C. The number of colonies was counted by using a Leica DM4000B microscope (Leica, Wetzlar, Germany).

### Cell cycle assay

6 × 10^5^ transfected Hep-2 and TU212 cells were seeded in 6-well plates and cultured for 48 h. Then, cells were washed twice with PBS and then resuspended with PBS. After that, cells were added 70% cold ethanol, mixed and incubated at 4°C overnight to fix the cell. The next day, the cells were collected by centrifugation. Under dark conditions, each sample was added with 2 μl RNase and 300 μl of PI (propidium iodide) dye solution, mixed and incubated for 30 min at room temperature and a dark environment. The cell cycle was detected by flow cytometry (EPICS, X1-4, Beckman, CA, U.S.A.).

### Wound healing assay

The migration ability of Hep-2 cells was determined by using wound healing assay. The transfected Hep-2 cells were seeded in 12-well plates and incubated in a humidified incubator at 37°C with 5% CO_2_ atmosphere overnight. when the cells were grown to 100% confluency, 100 μl plastic tips were applied to scrape three clear lines across the monolayer in each well. The debris was removed by washing three times with PBS, and cells were cultured in fresh prepared RPMI 1640 medium in a humidified incubator at 37°C. The width of the wounds was observed at different time points, and images were captured by using a Leica DM4000B microscope (Leica, Wetzlar, Germany).

### Transwell invasion assay

Before TU212 cells were seeded, Corning Costar Transwell 24-well plates (Corning, Corning, NY, U.S.A.) with Matrigel were placed in RPMI 1640 medium for 1 h at 37°C. A total of 2 × 10^4^ transfected cells were seeded in the wells, and were cultured in 500 µl RPMI 1640 medium without FBS. 500 µl RPMI 1640 medium containing 10% FBS was placed in the bottom wells. After incubation for 48 h at 37°C, the cells that did not migration through the pores were carefully removed by cotton swabs. Then, the invasive cells were fixed with methanol (Sigma–Aldrich, MO, U.S.A.) and stained with 0.1% crystal violet. Images were captured and number of invasive cells was counted under a Leica DM4000B microscope.

### Cell apoptosis assay

For cell apoptosis assay, 6 × 10^5^ transfected Hep-2 cells were seeded on 6-well plates for 48 h in a humidified chamber with 5% CO_2_ at 37°C. Then, cells were collected and washed three times with cold PBS, and resuspended with 1× binding solution (BD Biosciences, CA, U.S.A.) and stained with 5 μl Annexin V-FITC (BD Biosciences, CA, U.S.A.) and 3 μl PI (BD Biosciences, CA, U.S.A.). The percentage of apoptotic cells was detected by using flow cytometry.

### Caspase-3 activity assay

The apoptosis of TU212 cells was also detected by using Caspase-3 activity assay. Briefly, the transfected cells were seeded into 6-well plates (6×10^5^ cells/well) and cultured for 48 h. The apoptosis of cells was determined by using a Caspase-3 Colorimetric Assay Kit (BD Biosciences, CA, U.S.A.), according to the manufacturer’s protocols.

### Statistical analysis

Statistical analysis was analyzed by using SPSS statistical software, version 19.0 (SPSS, Chicago, IL, U.S.A.). All data were presented as mean ± SD (standard deviation) from at least three independent experiments. The association of FTH1P3 with clinicopathological features of LSCC patients was evaluated by Chi-square test. The overall survival of LSCC patients was generated by using the Kaplan–Meier method and was compared by using the log-rank test. The CCK-8 assay was analyzed by using by one-way analysis of variance (ANOVA) followed by Dunnett’s test. The Student’s *t*-test was used to analyze other data. A *P* value of less than 0.05 was considered statistically significant.

## Results

### FTH1P3 expression is up-regulated and correlates with malignant clinicopathological features in LSCC

**Figure 1:**
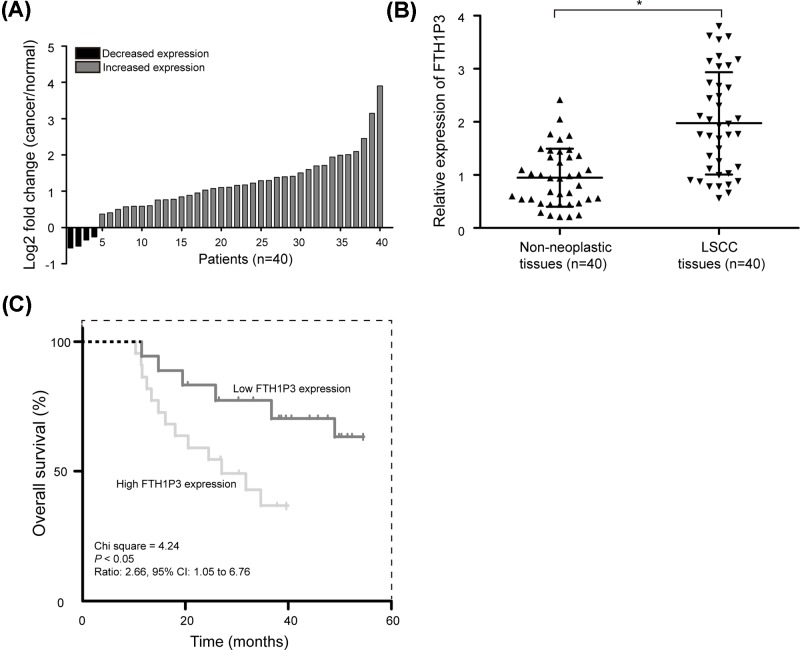
FTH1P3 expression is up-regulated and associates with poor overall survival in LSCC patients (**A**) qRT-PCR was utilized to detect relative levels of FTH1P3 in LSCC tissues (cancer) and non-neoplastic tissues (normal). (**B**) FTH1P3 expression was significantly increased in LSCC tissues compared with non-neoplastic tissues. (**C**) Kaplan–Meier analysis was performed for overall survival. LSCC patients with high FTH1P3 expression exhibited significantly poorer overall survival rates than those patients with low FTH1P3 expression as defined by log-rank test. *P<0.05.

Previous reports have shown that FTH1P3 expression is increased in LSCC tissues as determined by lncRNA microarray analysis [18]. To confirm the result of lncRNA profile study, qRT-PCR was used to detect the FTH1P3 expression in 40 LSCC patients. As shown in Figure 1A, among them, 36 cases (90.0%) showed increased expression levels of FTH1P3 in LSCC tissues compared with non-neoplastic tissues. Date analysis validated previous findings, and found that FTH1P3 levels were significantly higher (2.09-fold) in LSCC tissues than those in non-neoplastic tissues (Figure 1B, *P*<0.05).

Based on the expression data of FTH1P3 in LSCC tissues according to Youden’s index, we categorized 40 LSCC patients into low (*n*=22) and high (*n*=18) FTH1P3 expression groups. We determined whether the expression level of FTH1P3 was correlated with the clinicopathological features of LSCC patients, including gender, age, primary location, T classification, differentiation, lymph node metastasis, and clinical stage. As shown in Table 1, high FTH1P3 expression was positively correlated with the poor differentiation, high T classification, positive lymph node metastasis, and advanced clinical stage (*P*<0.05). But gender, age, and primary location had no associations with FTH1P3 expression. These results indicated that FTH1P3 is up-regulated and is positively correlated with malignant clinicopathological features of in LSCC.

### High FTH1P3 expression is associated with poor overall survival in LSCC patients

To further evaluate the clinical significance of FTH1P3 expression in LSCC, the survival curve was used to compare the difference in the overall survival rate between low and high FTH1P3 expression groups. With Kaplan–Meier method and log-rank test, we found that patients with high FTH1P3 expression had significantly poorer overall survival rates than those patients with low FTH1P3 expression (Figure 1C, *P*<0.05).

### FTH1P3 promotes cell proliferation in LSCC cells

**Figure 2:**
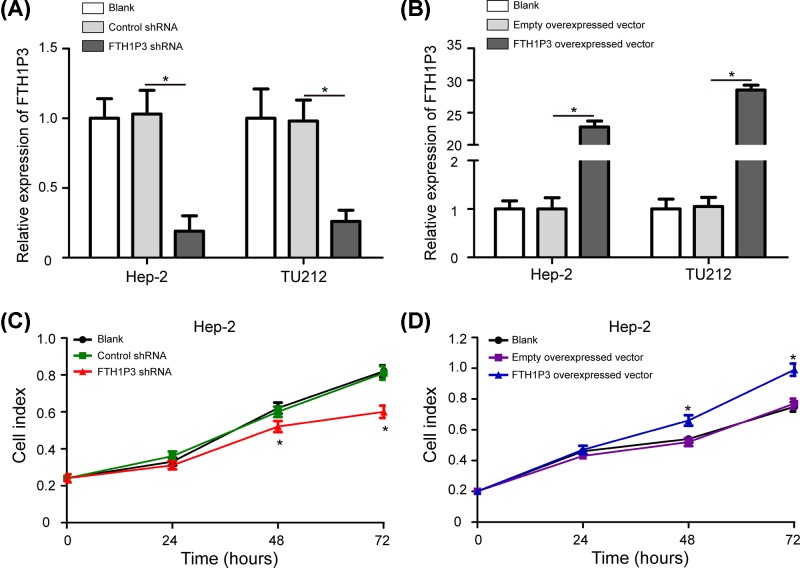
FTH1P3 promotes cell proliferation in LSCC cells (**A**) The expression changes of FTH1P3 in Hep-2 and TU212 cells after transfection of the FTH1P3 shRNA. The FTH1P3 shRNA significantly down-regulated the expression levels of FTH1P3 in Hep-2 and TU212 cells compared with the control shRNA group and blank group. (**B**) The FTH1P3 overexpressed vector remarkably up-regulated the expression levels of FTH1P3 in Hep-2 and TU212 cells compared with the empty overexpressed vector group and blank group. (**C**) CCK-8 assays were used to detect cell proliferation in Hep-2 cells by transfecting the FTH1P3 shRNA and control shRNA. (**D**) Hep-2 cells with the FTH1P3 overexpressed vector proliferated at a higher rate than the empty overexpressed vector group and blank group. FTH1P3 shRNA, FTH1P3 short hairpin RNA lentiviral vector; control shRNA, control shRNA lentiviral vector; FTH1P3 overexpressed vector, FTH1P3 overexpressed lentiviral vector; empty overexpressed vector, empty overexpressed lentiviral vector. *P<0.05.

Hep-2 and TU212 cells were cultured and then we inhibited FTH1P3 expression by transfecting the FTH1P3 shRNA and enhanced FTH1P3 expression by transfecting the FTH1P3 overexpressed vector. At 48 h after transfection, the related expression levels of FTH1P3 in Hep-2 and TU212 cells were determined by qRT-PCR analysis and the results showed that FTH1P3 expression in Hep-2 and TU212 cells was significantly down-regulated by the FTH1P3 shRNA compared with the control shRNA group and blank group (Figure 2A, *P*<0.05), whereas FTH1P3 level was dramatically up-regulated by the FTH1P3 overexpressed vector compared with the empty overexpressed vector group and blank group (Figure 2B, *P*<0.05).

We further determined whether FTH1P3 promoted cell proliferation in LSCC. Hep-2 cells were chose and transfected with the FTH1P3 shRNA or the FTH1P3 overexpressed vector, and cell proliferation abilities were determined by using both CCK-8 and colony formation assays. Decreased cell proliferation by silencing FTH1P3 was observed in Hep-2 cells as expected (Figure 2C, *P*<0.05). Hep-2 cells with the FTH1P3 overexpressed vector proliferated at a higher rate than the empty overexpressed vector group and blank group (Figure 2D, *P*<0.05).

**Figure 3:**
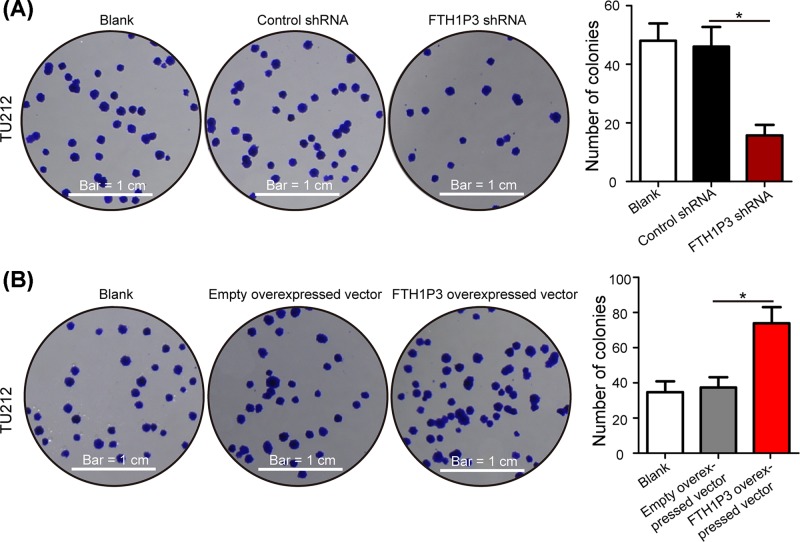
FTH1P3 promotes colony formation in LSCC cells (**A**) Colony formation assays were performed to detect TU212 cell proliferation after treatment with the FTH1P3 shRNA and control shRNA. (**B**) Overexpression of FTH1P3 dramatically promoted the abilities of colony formation in TU212 cells. Scale bar: 1 cm. *P<0.05.

**Figure 4:**
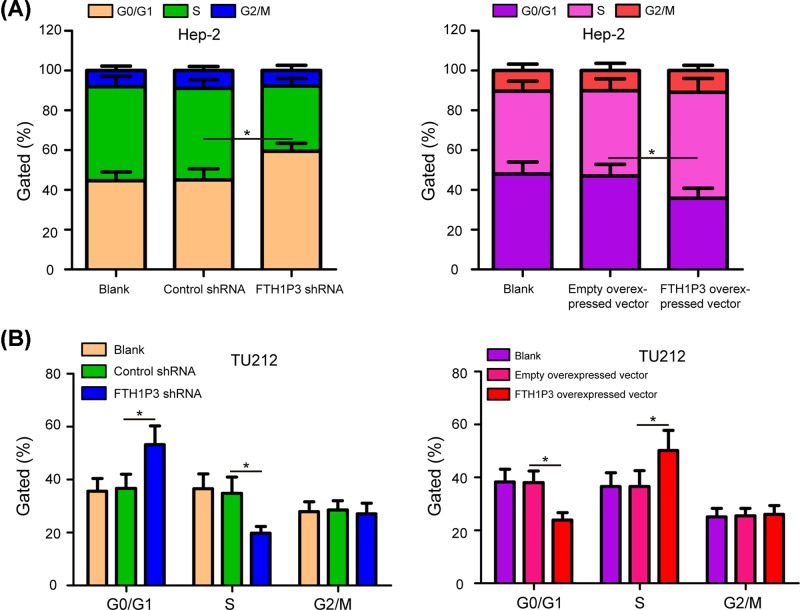
FTH1P3 promotes cell cycle progression in LSCC cells (**A**) Flow cytometry assays were used to measure the proportion of Hep-2 cells in each cell cycle phase. (**B**) Overexpression of FTH1P3 resulted in an increase in cells in S phase and a decrease in cells in G0/G1 phase in TU212 cells, whereas inhibition of FTH1P3 did the opposite effects. *P<0.05.

Subsequently, colony formation assays were also illustrated to detect cell proliferation. As shown in Figure 3A, compared with the control shRNA group and blank group, the number of colonies in TU212 cells were reduced after treatment with the FTH1P3 shRNA (*P*<0.05). Compared with the empty overexpressed vector group and blank group, the number of colonies was obviously increased in TU212 cells transfected with the FTH1P3 overexpressed vector (Figure 3B, *P*<0.05). To assess mechanism of FTH1P3 affecting LSCC cell proliferation, the experiment of cell cycle was performed in Hep-2 and TU212 cells. The data showed that inhibition of FTH1P3 resulted in a decrease in Hep-2 cells in S phase and an increase in cells in G0/G1 phase, whereas overexpression of FTH1P3 did the opposite effects (Figure 4A, *P*<0.05). In addition, similar results of cell cycle were also found in TU212 cells (Figure 4B, *P*<0.05). Taken together, our results demonstrated that FTH1P3 promotes proliferation of LSCC cells.

### FTH1P3 accelerates cell migration and invasion in LSCC cells

**Figure 5:**
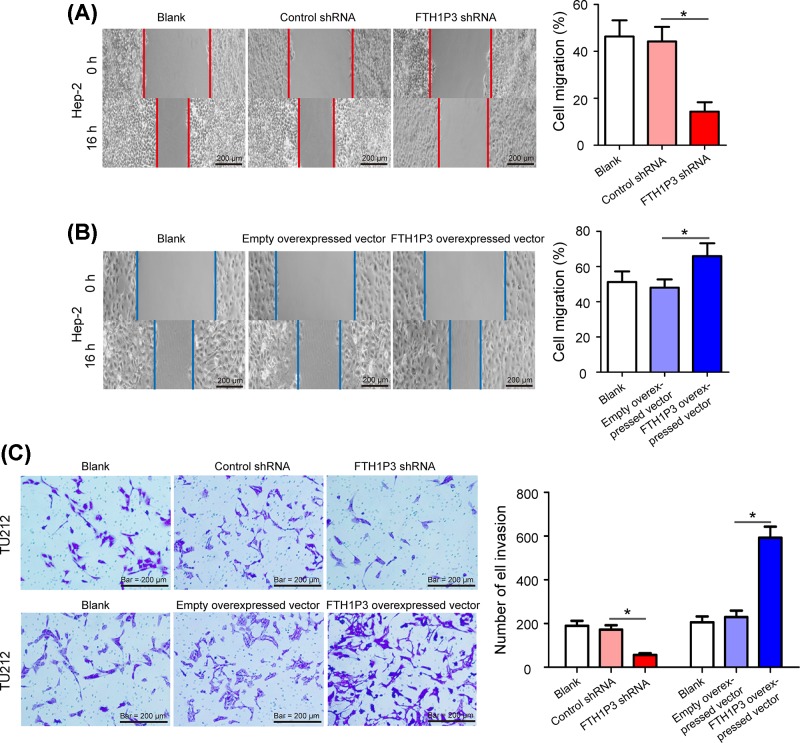
FTH1P3 accelerates cell migration and invasion in LSCC cells (**A**) Cell migration of Hep-2 cells was significantly inhibited in Hep-2 cells by transfecting the FTH1P3 shRNA. (**B**) Cell migration was promoted in Hep-2 cells by transfecting the FTH1P3 overexpressed vector. (**C**) Transwell invasion assays were performed in TU212 cells, silencing FTH1P3 resulted in reduced cell invasion, whereas elevated expression of FTH1P3 increased the invasion capacities of TU212 cells. Scale bar: 200 μm. *P<0.05.

Wound healing assays were utilized to detect the role of FTH1P3 in the migration of Hep-2 cells. Compared with the control shRNA group and blank group, the ratio of cell migration in the FTH1P3 shRNA group was significantly reduced in Hep-2 cells (Figure 5A, *P*<0.05). Compared with the empty overexpressed vector group and blank group, cell migration was promoted in Hep-2 cells by transfecting the FTH1P3 overexpressed vector (Figure 5B, *P*<0.05). Besides, transwell invasion assays also showed reduced cell invasion of the FTH1P3 shRNA transfected TU212 cells, and increased cell invasion in the FTH1P3 overexpressed vector group (Figure 5C, *P*<0.05). These data strongly suggested that FTH1P3 accelerates cell migration and invasion of LSCC cells.

### FTH1P3 inhibits cell apoptosis in LSCC cells

**Figure 6:**
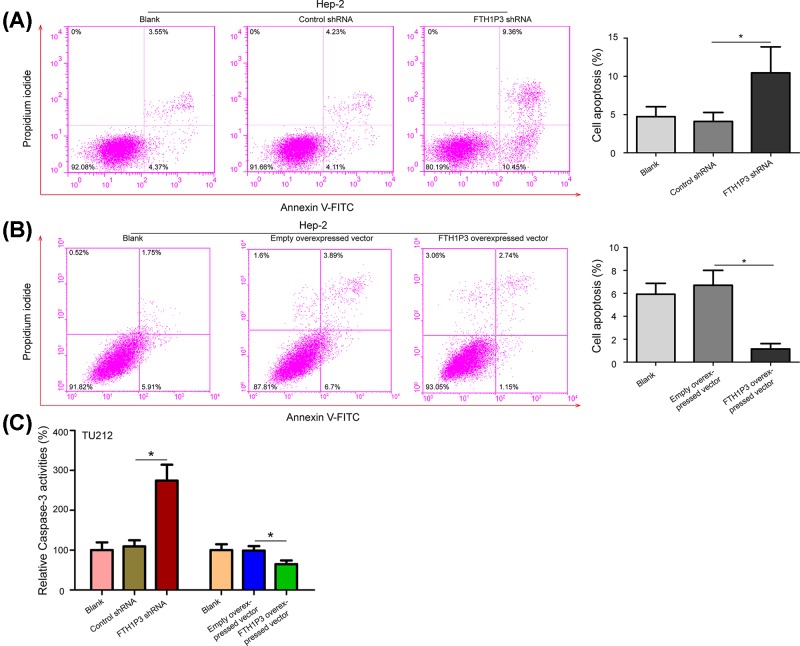
FTH1P3 inhibits cell apoptosis in LSCC cells (**A**) The apoptotic cells were observed in Hep-2 cells treated with the FTH1P3 shRNA and control shRNA. (**B**) Cell apoptosis inhibition was observed in the FTH1P3 overexpressed vector compared with the empty overexpressed vector group and blank group. (**C**) Caspase-3 activity assay was also performed to detected the effects of FTH1P3 silencing and overexpression on cell apoptosis in TU212 cells. *P<0.05.

We further determined whether FTH1P3 suppressed cell apoptosis in LSCC. Hep-2 and TU212 cells were transfected with the FTH1P3 shRNA or the FTH1P3 overexpressed vector, and cell apoptosis changes were determined by using flow cytometry and Caspase-3 activity assays. Interesting, induced cell apoptosis by silencing FTH1P3 was observed in Hep-2 cells. The ratio of apoptosis in the FTH1P3 shRNA group was increased 1.54-fold compared with the control shRNA group and blank group (Figure 6A, *P*<0.05). Suppressed cell apoptosis by transfecting the FTH1P3 overexpressed vector was observed in Hep-2 cells. The ratio of apoptosis in the FTH1P3 overexpressed vector group was decreased 0.83-fold compared with the empty overexpressed vector group and blank group (Figure 6B, *P*<0.05). In addition, Caspase-3 activity assays showed that the activities of Caspase-3 in the FTH1P3 shRNA group were increased, while the activities of Caspase-3 in the FTH1P3 overexpressed group were decreased (Figure 6C, *P*<0.05). These results indicate that FTH1P3 reduces cell apoptosis of LSCC cells.

## Discussion

The development of LSCC involves various genetic and epigenetic machineries [20]. Because there are no specific symptoms at the early stage of LSCC, most LSCC patients are found at advanced stage, and treatments are less effective. LncRNAs are a class of noncoding RNA transcripts with no protein-coding capacity. Recent studies have illustrated that lncRNAs play key regulatory roles in carcinogenesis [21]. LncRNAs are involved in multiple processes of tumorigenesis via regulating gene expression by a variety of mechanisms, including transcription, post-transcription, chromatin remodeling, and protein modification [22,23]. Therefore, in-depth understanding of lncRNAs may be helpful in identification of novel effective therapeutic strategies for the treatment of LSCC.

With recent advancement of the knowledge of lncRNAs, a number of lncRNAs have been shown to participate in LSCC progression. For instance, Wu et al. [24] reported that lncRNA H19 (imprinted maternally expressed transcript H19) promotes LSCC progression via targeting microRNA-148a-3p and DNMT1 (DNA methyltransferase 1). Yang and co-workers [25] showed that lncRNA HOXA11-AS (HOXA11 antisense RNA) plays an oncogenic role in the cellular processes of LSCC. Zhang and Hu [26] found that lncRNA CCAT1 (Colon cancer associated transcript 1) stimulates the progression of LSCC by targeting microRNA-218/ZFX axis. FTH1P3 is one of the ferritin heavy polypeptide intronless copies located on chromosome 2. But there is no report about the relationship between FTH1P3 and LSCC yet.

Previous documents have found that FTH1P3 is up-regulated in OSCC and uveal melanoma, and promotes tumor progression [16,17]. Microarray data of lncRNAs from Shen et al. showed that FTH1P3 expression is also up-regulated in LSCC [18]. In the present study, by qRT-PCR assay we confirmed that FTH1P3 expression was significantly higher in LSCC tissues than that in non-neoplastic tissues. High FTH1P3 expression was positively correlated with poor differentiation, high T classification, positive lymph node metastasis, and advanced clinical stage. Moreover, patients with high FTH1P3 expression had significantly poorer overall survival rates compared with those patients with low FTH1P3 expression. These results indicated that high level of FTH1P3 is associated with the malignant phenotypes of LSCC patients.

Previous literature has found that FTH1P3 promoted cell proliferation and invasion in uveal melanoma [17]. In order to understand the biological functions of FTH1P3 in LSCC cells, we detected the cell proliferation, migration, invasion, apoptosis, and cell cycle by silencing and overexpressing FTH1P3 in Hep-2 and TU212 cells. Similarly, our study showed that FTH1P3 silencing exerted inhibitory effects on the proliferation, migration and invasion of Hep-2 and TU212 cells, and played promotive effect on cell apoptosis. Conversely, overexpression of FTH1P3 promoted LSCC cell proliferation, migration and invasion, and inhibited cell apoptosis. In addition, overexpression of FTH1P3 resulted in an increase in cells in S phase and a decrease in cells in G0/G1 phase, whereas inhibition of FTH1P3 did the opposite effects. These data suggested that FTH1P3 could act as an oncogene to promote the progression of LSCC, providing a promising therapeutic target for the treatment of LSCC.

In conclusion, this research elucidated that FTH1P3 expression is up-regulated in LSCC tissues and associates with malignant clinicopathological features in LSCC. To our knowledge, this is the first study of FTH1P3 playing an oncogenic role in the progression of LSCC. FTH1P3 may be a promising biomarker for LSCC treatment. More work will be needed to determine the molecular mechanisms of FTH1P3 in LSCC.

## Availability of data and materials

The analyzed data sets generated during the study are available from the corresponding author on reasonable request.

## Abbreviations

CCK-8: cell count kit-8
FTH1P3: ferritin heavy chain 1 pseudogene 3
lncRNA: long noncoding RNA
LSCC: laryngeal squamous cell carcinoma
PI: propidium iodide
qRT-PCR: quantitative real-time PCR
